# Supplementary figures and images for: Structure-based identification of potent fibroblast growth factor receptor 4 (FGFR4) inhibitors as potential therapeutics for hepatocellular carcinoma
Source: PeerJ. 2025 Jun 18;13:e19183. doi: 10.7717/peerj.19183 (PMC12182054; doi:10.7717/peerj.19183)

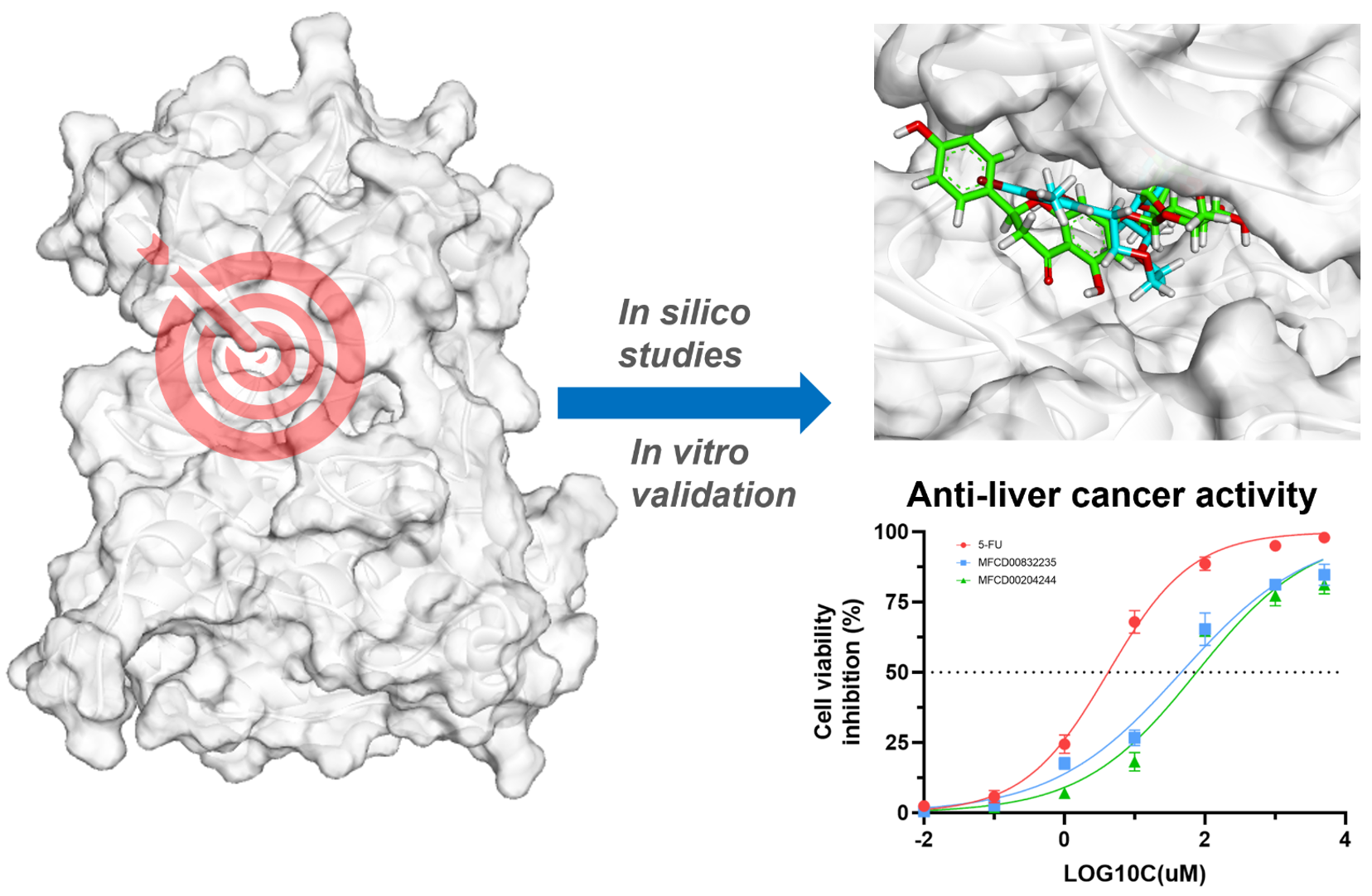

Supplement: Supplemental Information 2 — Both compounds exhibited high dynamic stability, low reactivity, and favorable binding to FGFR4, with in vitro assays demonstrating their potential to inhibit HepG2 cell growth. [file peerj-13-19183-s002.png]
